# Identification of *LDLR* mutation in cerebral venous sinus thrombosis co-existing with dural arteriovenous fistulas: a case report

**DOI:** 10.1186/s12883-023-03455-5

**Published:** 2023-11-28

**Authors:** Qing-hua Li, Li-quan Xu, Qiang Dong, He-ling Chu, Yu-ping Tang

**Affiliations:** 1 Department of Neurology, Hunan Provincial People’s Hospital, No. 61 Jiefang West Road, Furong District, Changsha, Hunan Province 410005 China; 2 Department of Neurosurgery, Fudan University Affiliated Huashan Hospital, No. 12 Wulumuqi Road, Shanghai, 200040 China; 3 Department of Neurology, Fudan University Affiliated Huashan Hospital, No. 12 Wulumuqi Road, Shanghai, 200040 China; 4 Department of Gerontology, Shanghai Sixth People’s Hospital Affiliated to Shanghai Jiao Tong University School of Medicine, No. 600 Yishan Road, Shanghai, 200233 China

**Keywords:** Dural arteriovenous fistulas, Cerebral venous sinus Thrombosis, Low-density lipoprotein receptor, Gene mutation, Case report

## Abstract

**Background:**

Cerebral venous sinus thrombosis (CVST) is typically associated with a prothrombotic state of the blood, with its causative factors varying widely. Prior research has not reported the simultaneous occurrence of CVST and dural arteriovenous fistulas (DAVFs) as potentially resulting from genetic mutations. In this case report, we introduce a unique occurrence wherein a patient with a heterozygous mutation of the low-density lipoprotein receptor (*LDLR)* gene presented with CVST in conjunction with DAVFs.

**Case:**

P**resentation**: A male patient, aged 51, sought treatment at our facility due to a consistent decline in cognitive functions accompanied by recurrent headaches. Comprehensive evaluations were administered, including neurological examinations, laboratory tests, magnetic resonance imaging, digital subtraction angiography, and whole exome sequencing. Digital subtraction angiography identified DAVFs in the patient’s right sigmoid sinus and an occlusion within the left transverse sinus. The whole exome sequencing of blood samples pinpointed a heterozygous mutation in the *LDLR* gene (NM_000527:exon12:c.C1747T:p.H583Y). Following the confirmed diagnosis of CVST and DAVFs, the patient underwent anticoagulant therapy combined with endovascular procedures — these comprised embolization of the arteriovenous fistula in the right sigmoid sinus and balloon dilation with stent implantation in the left transverse sinus. A six-month follow-up indicated a significant abatement in the patient’s symptoms.

**Conclusions:**

This report marks the first documented case of an *LDLR* gene mutation that could be associated with the onset of CVST and DAVFs. The mutation in the *LDLR* gene might foster a prothrombotic environment, facilitating the gradual emergence of CVST and the subsequent genesis of DAVFs.

## Background

Cerebral venous sinus thrombosis (CVST) and dural arteriovenous fistula (DAVFs) are both rare cerebrovascular diseases. The co-existence of CVST and DAVFs is even rarer. Previous clinical studies have suggested that CVST is closely associated with DAVFs and may account for the subsequent occurrence of DAVFs. CVST can result from inherited thrombophilia, an inherited predisposition to thrombosis, which includes factor V Leiden mutation, protein C and S deficiency, sickle cell disease, and prothrombin 20,210 gene mutation [1]. Here, we present a patient who carried a heterozygous mutation in the low-density lipoprotein receptor (*LDLR)* gene and suffered from both CVST and DAVFs.

## Case presentation

### Patient information

A 51-year-old male patient presented at our hospital, reporting a decline in cognitive functions and notable personality alterations over the previous five months. Throughout this timeframe, he exhibited symptoms consistent with dementia, such as memory deficits, growing apathy, and a decline in his professional performance as a driver. The patient had a background of non-specific headaches. Over time, his condition worsened, manifesting in an increasingly irregular walking pattern and involuntary tremors of the upper limbs when engaging in tasks like using chopsticks or holding items.

### Neurological examinations

This patient was conscious and spoke fluently during neurological examinations. Both pupils were normal, equal in size, and reactive to light. No nuchal rigidity or other signs of meningitis were identified. The cranial nerve, motor, sensory examinations and reflexes showed no abnormalities. He had a positive Romberg test and postural and intentional tremors in his upper limbs. The cognitive assessment revealed severe memory, orientation, comprehension, and calculation impairments. He scored 6 out of 30 on the mini-mental state examination (MMSE) and 3 out of 30 on the Montreal Cognitive Assessment (MoCA).

### Laboratory data

Blood tests, including blood cell analysis, thyroid hormones, vitamin B12, folic acid, treponema pallidum particle agglutination, and human immunodeficiency virus, excluded some reversible causes of cognitive impairment. Biochemical tests showed no abnormalities. Lupus anticoagulant, erythrocyte sedimentation rate, rheumatoid factor, antineutrophil cytoplasmic antibody, anti-double-stranded DNA antibody, antinuclear antibodies, and antiphospholipid antibodies were normal. Homocysteine, coagulation function, D-dimer, protein C, protein S, and antithrombin III were also within the normal range.

Lumbar puncture examination revealed that the cerebrospinal fluid (CSF) pressure was 280 mmH_2_O. The total protein level was 0.56 µg/L (normal range: 0.15–0.45 µg/L). CSF cell count, glucose, electrolytes, and lactate dehydrogenase were within the normal range. Microbiological tests showed no abnormalities. Autoimmune encephalitis antibodies in both the CSF and serum were negative.

### Imaging data

**Fig. 1:**
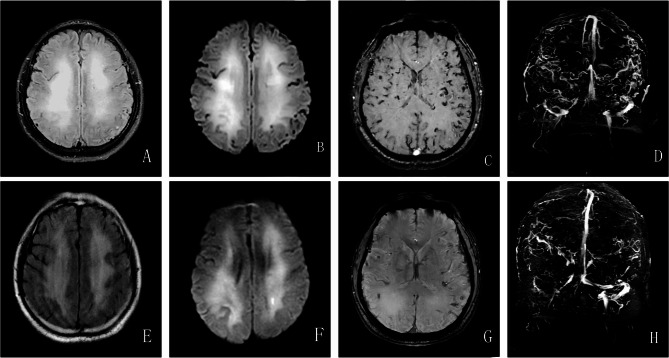
Brain MRI scan. T2-weighted FLAIR (**A**) and DWI (**B**) sequences of brain MRI scans reveal abnormal white matter signals in bilateral corona radiata areas. SWI (**C**) and MRV (**D**) sequences of brain MRI scans reveal extensive tortuous dilatation of intracranial veins. After DAVF embolization, T2-weighted FLAIR (**E**) and DWI (**F**) brain MRI scans show improvement in white matter lesions. SWI (**G**) and MRV (**H**) sequences of brain MRI show a significant reduction in multiple vascular shadows on the brain surface and subcortex. Abbreviations: MRI = magnetic resonance imaging; FLAIR = fluid attenuation inversion recovery; DWI = diffusion-weighted imaging; SWI = susceptibility-weighted imaging; MRV = magnetic resonance venography; DAVF = dural arteriovenous fistula

Brain magnetic resonance imaging (MRI) displayed diffuse T2-weighted fluid-attenuated inversion recovery (FLAIR) and diffusion-weighted imaging (DWI) white matter hyperintensities in bilateral corona radiata areas (Fig. 1A-B). The susceptibility-weighted imaging (SWI) and magnetic resonance venogram (MRV) of the brain showed extensive tortuous dilatation of the intracranial veins (Fig. 1C-D).

**Fig. 2:**
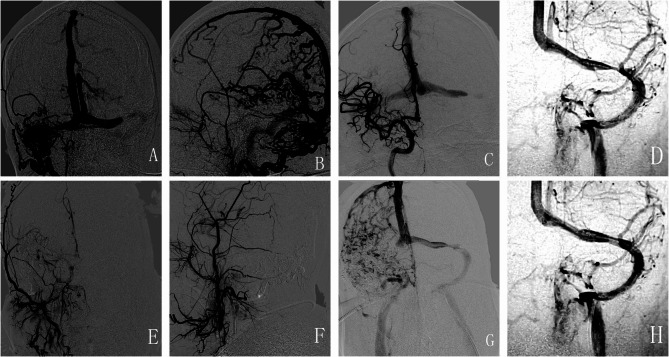
DSA images. In the frontal view (**A**) and lateral view (**B**), preoperative DSA shows a DAVF in the right sigmoid sinus and occlusion in the left transverse sinus (**C**) (arrows). After endovascular embolization therapy, the frontal view (**E**) and lateral view (**F**) of DSA indicate complete occlusion of the DAVF. Following the first balloon dilation, the left transverse (arrow) sinus occlusion was partially recanalized (**G**). After the second balloon dilation (**D**) and stent implantation (**H**), DSA shows a resolution of left transverse sinus stenosis (arrows). Abbreviations: DSA = digital subtraction angiography

Digital subtraction angiography (DSA) revealed a DAVF in the right sigmoid sinus (Fig. 2A-B) and CVST of the left transverse sinus (Fig. 2C). The DAVF was fed from the right occipital artery and middle meningeal artery.

### Gene detection data

**Fig. 3:**
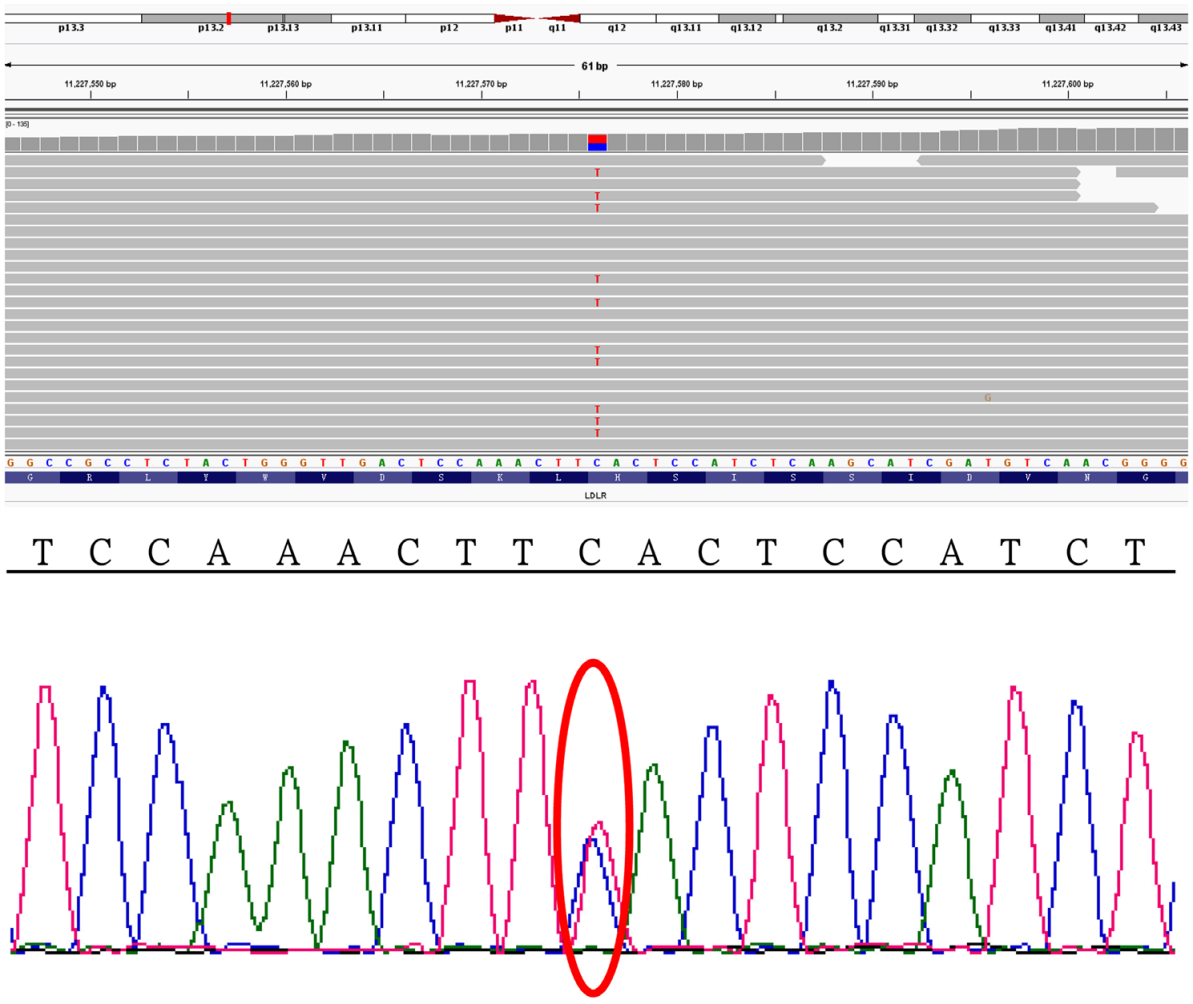
Whole exome sequencing shows a heterozygous mutation in the *LDLR* gene (NM_000527: exon12:c.C1747T:p.H583Y).

Whole exome sequencing of blood samples revealed a heterozygous mutation in exon 12 of the LDLR gene (c.C1747T), resulting in an amino acid change from histidine to tyrosine (p.H583Y) (Fig. 3).

### Endovascular procedures

Angiography revealed the right sigmoid sinus DAVF; the fistula was fed by bilateral occipital arteries, meningeal hypophyseal trunks, middle meningeal arteries, etc.; meanwhile, the left sigmoid sinus was occluded.

The procedure was performed under general heparinization. Transarterial embolization was performed via the meningeal branch of the right posterior cerebral artery, and 0.2 ml Onyx-18 was injected gently to occlude the pial feeding artery. Then, the Marathon microcatheter was advanced via the meningeal branch of the right occipital artery, and 6.5 ml Onyx-18 was injected, obliterating the fistula.

The 8 F Britetip catheter was advanced to the proximal side of the occlude left sigmoid sinus via a transvenous access route. The microcatheter was penetrated to the superior sagittal sinus. The occluded segment was expanded with a Gateway 4.0*9mm (Boston Scientific) and a Stering 5.0*20 (Boston Scientific) balloon sequentially over the Floppy-300 exchanging wire. Direct venography revealed moderate residual sinus stenosis. Post-operative Dyna-CT demonstrated no hemorrhage.

A Wallstent 7.0*30mm stent (Boston scientific) was deployed at the stenotic sinus in the second stage. After sinus stenting, the cerebral antegrade drainage improved significantly.

### Diagnosis, treatment, and follow-up

Shortly after admission, the diagnoses of CVST and DAVFs were made, and the patient received oral anticoagulation treatment with rivaroxaban. Subsequently, DAVF embolization (Fig. 2E-F) and balloon dilation of the left transverse sinus (Fig. 2G) were performed. Ten days after the surgery, a brain MRI showed that white matter hyperintensity decreased on T2-weighted FLAIR and DWI sequences (Fig. 1E-F). SWI and MRV imaging showed a significant reduction in multiple vascular shadows on the brain surface and subcortex (Fig. 1G-H).

Two months later, the MMSE score of this patient was 18 out of 30 and the MoCA score was 10 out of 30, suggesting significant cognitive function improvement after treatment. However, his CSF pressure remained above 200 mmH_2_O despite ongoing anticoagulant therapy. Therefore, three months after the first endovascular therapy, this patient underwent another balloon dilatation (Fig. 2D) and stent implantation for severe stenosis in the left transverse sinus (Fig. 2H). After the surgery, dual antiplatelet therapy with aspirin and clopidogrel was administered for three months to prevent stent thrombosis. In the telephone follow-up conducted six months later, the patient’s cognitive function improved significantly, and he demonstrated a better ability to perform daily life activities. He could also take care of himself without any walking disruptions. Given the association between CVST complicated with DAVFs and the *LDLR* gene mutation, continuous anticoagulation therapy with rivaroxaban was recommended [2].

## Discussion and conclusions

The swift advancement of dementia is a rare neurological manifestation, typically necessitating an exhaustive assessment, especially for potentially reversible causes. In the case of this 51-year-old male patient who presented with progressive dementia, his brain MRI revealed bilateral diffuse white matter hyperintensity across the cerebral hemispheres. A plausible pathogenic mechanism suggests that the DAVFs in the right sigmoid sinus and an occlusion in the left transverse sinus increase venous pressure. This elevation in pressure might contribute to venous hypertensive encephalopathy and the rapid deterioration of cognitive abilities, as documented in prior reports [3].

DAVFs, referring to pathologic shunts between meningeal arteries and dural venous sinuses or veins, are potentially associated with CVST. However, it remains unclear whether DAVFs cause CVST or develop as a result of CVST [1, 3]. In this case, follow-up interviews indicated that despite early embolization of DAVFs, the patient’s intracranial pressure remained elevated due to CVST. These observations suggest that CVST might lead to the formation of DAVFs.

CVST is a rare clinical condition with various predisposing factors. Over 85% of the diagnosed cases have at least one identified risk factor, including acquired and genetic prothrombotic conditions. Common acquired risks include neurosurgical procedures, adjacent infections, trauma, pregnancy, puerperium, antiphospholipid syndrome, obesity, malignancy, exogenous hormones, oral contraception, and COVID-19. The genetic risks refer to inherited thrombophilia conditions, such as hyperhomocysteinemia, antithrombin III deficiency, protein C or protein S deficiency, factor V Leiden mutation, and G20210A prothrombin gene mutation. The cause of CVST remains unidentified in approximately 15% of patients. Identifying the exact cause of CVST in each patient is essential but complex. No clear etiologies or risk factors for CVST were initially found in this case. Then, whole genome exome sequencing revealed a heterozygous mutation in the *LDLR* gene (NM_000527:exon12:c.C1747T:p.H583Y). To our knowledge, this is the first report of an *LDLR* gene mutation possibly associated with the development of CVST and subsequent DAVFs.

Previous studies have demonstrated a significant correlation between LDLR gene mutations and coronary and cerebral artery thrombosis [4, 5]. The relationship between the *LDLR* mutation and venous thrombosis has also been reported. A whole exome sequencing study conducted on patients with sickle cell disease identified a patient with heterozygous mutations in the *LDLR* gene who also had a history of deep venous thrombosis [6]. The *LDLR* mutations in the patient led to altered expression of specific proteins associated with thrombosis, including factor VIII and anticardiolipin antibodies. Increasing evidence has shown that a high plasma level of factor VIII is an independent risk factor for venous thrombosis [7]. Kyrle et al. showed that factor VIII levels exceeding 150 mcg/L are associated with a 5-fold increase in the risk of venous thrombosis [8]. Furthermore, Bovenschen et al. observed an approximately 4.2-fold rise in factor VIII levels in *LDLR*-deficient mice [9]. LDLR has also been identified as contributing to factor VIII homeostasis [9]. Additionally, Ochoa et al. showed that individuals carrying the anticardiolipin antibody exhibited a significantly lower LDLR gene expression level than the control group [10].

In conclusion, this case report posits that the mutation of the *LDLR* gene could induce a prothrombotic state, potentially facilitating the gradual onset of CVST and the ensuing formation of DAVFs. More extensive research is essential to determine the relative risk and prevalence of *LDLR* gene mutations in patients diagnosed with DAVF and CVST. Given the paucity of existing literature on the correlation between LDLR mutations and CVST and the absence of related animal studies, our findings call for validation through more comprehensive investigations, encompassing both animal experiments and clinical studies.

## Data Availability

The data and materials used or analyzed during the current study are available from the corresponding author upon reasonable request.

## Abbreviations

DAVFs: Dural arteriovenous fistulas
CVST: Cerebral venous sinus thrombosis
LDLR: Low-density lipoprotein receptor
MMSE: Mini-mental state examination
MoCA: Montreal Cognitive Assessment
FLAIR: Fluid attenuated inversion recovery
SWI: Susceptibility-weighted imaging
MRV: Magnetic resonance venogram
DSA: Digital subtraction angiography
